# All-Solid-State Ion-Selective Electrode Inspired from All-Solid-State Li-Ion Batteries

**DOI:** 10.1021/acs.analchem.4c06470

**Published:** 2025-02-26

**Authors:** Ryoichi Tatara, Yuki Shibasaki, Daisuke Igarashi, Hiroyuki Osada, Kazuma Aoki, Yusuke Miyamoto, Toshiharu Takayama, Takahiro Matsui, Shinichi Komaba

**Affiliations:** † Department of Applied Chemistry, Tokyo University of Science, 1-3 Kagurazaka, Shinjuku, Tokyo 162-8601, Japan; ‡ New Applications Research Center, KOA CORPORATION, 1634-17 minami-minowa, Kamiina, Nagano 399-4511, Japan

## Abstract

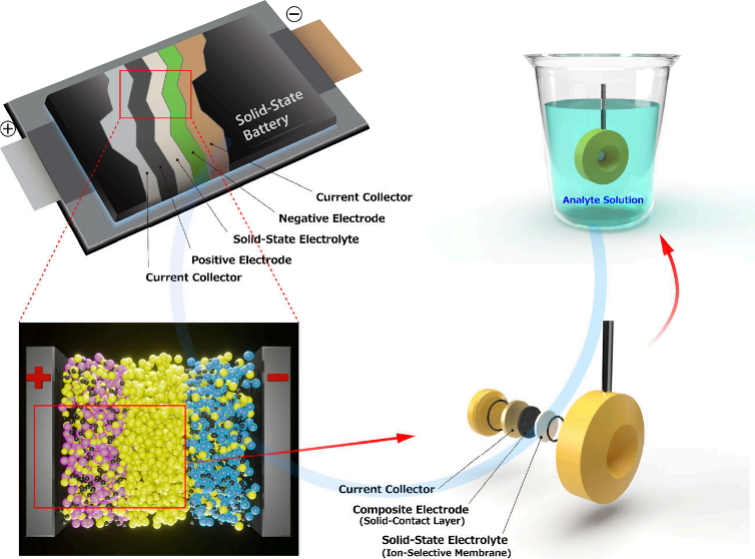

Solid electrolytes employed in all-solid-state Li-ion batteries (ASSBs) electronically isolate the positive and negative electrodes, while allowing the carrier ions, Li^+^, to pass through. Inorganic solid-state electrolytes, which typically exhibit a Li^+^-transference number of 1, are theoretically applicable as ion-sensitive membranes of potentiometric ion-selective electrodes (ISEs). Inspired by the ASSB architecture, an all-solid-state Li ISE was developed in a two-layer stacking configuration using a redox-active material (LiFePO_4_) and a solid electrolyte (Li_1+*x*+*y*_Al_*x*_(Ti, Ge)_2–*x*_Si_*y*_P_3–*y*_O_12_) as inner and outer layers, respectively, on the substrate (i.e., current collector). The solid electrolyte acts as an ion-selective membrane because the Donnan membrane potential obeys a Nernstian response to Li^+^ activity in the analyte solution. The fabricated ASSB-inspired ISE selectively responds to Li ions, exhibiting a Nernstian slope of 60.8 ± 0.5 mV dec^–1^, limit of detection of 10^–4.9±0.4^, and minimal potential variation (−3 to +6 mV over 17 d). Using a two-phase LiFePO_4_/FePO_4_ layer with a highly stable potential as the inner reference electrode significantly minimizes the deviations in the response potential. Moreover, applying Li_1+*x*+*y*_Al_*x*_(Ti, Ge)_2–*x*_Si_*y*_P_3–*y*_O_12_ as a durable and highly ion-conductive inorganic solid electrolyte enables remarkable long-term stability.

Ion-selective electrodes (ISEs) are a class of electrochemical sensors that are renowned for their ability to determine ion concentrations (more accurately, activity) in solutions with exceptional selectivity and sensitivity.^1−7^ ISEs play a critical role in various applications, such as food nutritional analysis,^8^ drainage analysis,^9^ and fertilizer concentration monitoring in large-scale hydroponic farming systems.^10^ Traditional pH sensors, which are essentially H^+^-ISE, utilize a glass membrane, whereas more recent ISEs for various ion species comprise an ion-selective membrane (ISM) that incorporates organic ionophores, an internal filling solution, and an internal reference electrode. Typically, ISEs are potentiometric ion sensors that are used to determine the concentration of a specific ion in an aqueous solution by gauging the membrane potential arising from the disparity in the ionic activities between the internal and the analyte solutions. Nevertheless, the conventional ISE design, which necessitates an internal reference electrode and internal solution, restricts device miniaturization.^11^ Therefore, novel all-solid-state ISEs, in which both the internal reference electrode and solution are substituted with electrode materials used in rechargeable batteries (forming a solid-contact layer), have been explored.^12−20^ However, these ISEs predominantly employ organic ionophores in the ISM. Previously, our group reported an all-solid-state ISE based on the combination of Li-, Na-, and K-ion battery materials and anion-insertion materials with organic membranes.^13,16,19,20^ On the other hand, inorganic solid electrolytes, integral to all-solid-state Li-ion batteries (ASSBs) and increasingly studied for their practical utility, exhibit a carrier ion transference number of 1^21^ and can potentially serve as effective ISMs. Notably, the assembly technique used in ASSBs^22^—integrating a solid electrolyte (i.e., an ISM) with an electrode material that serves as the reference electrode—can be adopted for ISEs. This paper presents an all-solid-state ISE inspired by all-solid-state battery design and discusses its performance.

Figure 1a,b schematically illustrates the fabricated ISE (the experimental details are provided in the Supporting Information). The solid contact layer consisted of a blend of LiFePO_4_, a conventional positive electrode material used in lithium-ion batteries, and FePO_4_ obtained by the chemical delithiation of LiFePO_4_ (that is, the chemical oxidation of LiFePO_4_ in a 0.1 M aqueous Na_2_S_2_O_8_ solution).^23^ This combination was selected to stabilize the potential and serve as an internal reference electrode, as reported previously.^16^ To mimic a typical lithium-ion battery composite electrode, acetylene black and poly(vinylidene difluoride) were incorporated, and a mixture of lithium bis(trifluoromethanesulfonyl)amide (LiTFSA) and poly(ethylene oxide) (PEO), which functions as a solid polymer electrolyte, was added to the composite electrode to ensure ion conduction.^24^ A 150-μm-thick plate of Li^+^-substituted Na superionic conductor (NASICON)-type commercial solid electrolyte, Li_1+*x*+*y*_Al_*x*_(Ti, Ge)_2–*x*_Si_*y*_P_3–*y*_O_12_ (hereafter denoted as LATP), was placed over the LiFePO_4_/FePO_4_ mixture. LATP is known for its stability, except in very acidic or basic environments.^25^ The fabricated ISE was aged overnight at 80 °C to melt PEO and thus improve the contact between the LATP and the LiFePO_4_/FePO_4_ layers. Hereafter, this ISE configuration is referred to as the solid battery (SB)-type ISE.

**Figure 1:**
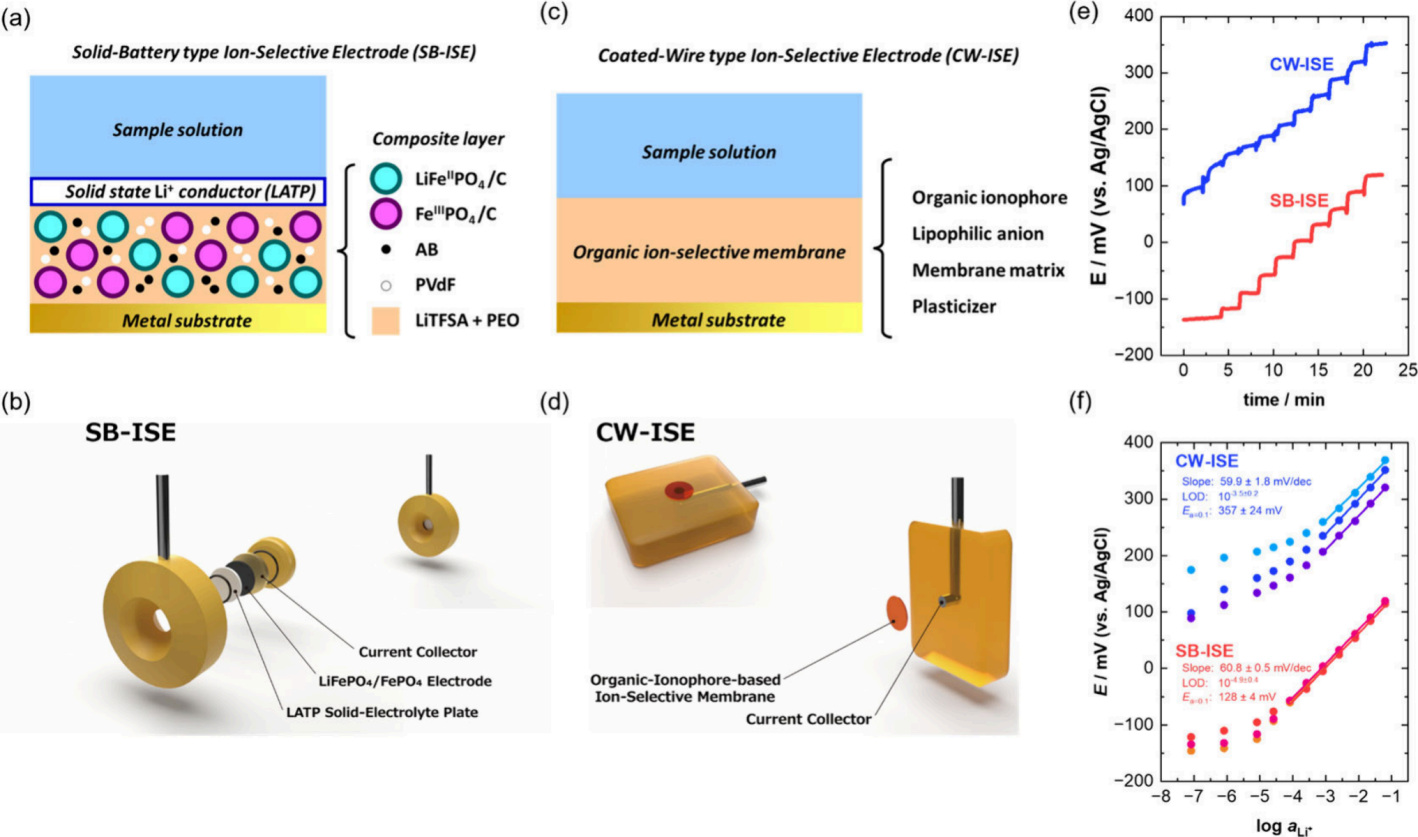
Schematic illustration of (a, b) solid-battery-type ion-selective electrode (SB-ISE) and (c, d) conventional coated-wire-type ion-selective electrode (CW-ISE). (e) Potential vs time curve recorded with the sequential addition of LiCl, and (f) calibration plots of the fabricated SB- and CW-ISEs obtained in an aqueous LiCl solution containing 0.01 mol dm^–3^ tris(hydroxymethyl)aminomethane as pH buffer versus a Ag/AgCl electrode in a saturated aqueous KCl solution. The LiCl concentration was converted to Li^+^ activity using the extended Debye–Hückel equation (Equation S1 and Table S1).^29^ Error values (±) are derived from the standard deviations.

For comparative analysis, Figure 1c,d illustrates a coated wire (CW)-type ISE prepared by directly forming an organic ionophore-based ISM on the electrode substrate.^11^Figure 1e shows the typical potential response of the ISE during a sequential change in the Li^+^ (LiCl) concentration, and Figure 1f displays Nernst plots (calibration curves) obtained using the CW- and SB-type ISEs (three electrodes each). Both the SB- and CW-type ISEs demonstrated prompt potential responses to changes in the Li-ion concentration. The CW-type ISE exhibited a Nernst slope of 59.9 ± 1.8 mV dec^–1^, while the SB-type ISE displayed a slope of 60.8 ± 0.5 mV dec^–1^, which are close to the theoretical value of 59.2 mV dec^–1^ at 25 °C. The limit of detection (LOD) of the SB-ISE is 10^–4.9±0.4^, which is adequate for analyzing food products and hydroponic solutions. Although the LOD of the CW-ISE is slightly higher than that of the SB-ISE, it is worth noting that numerous lithium ionophores have been developed recently. Lithium ionophore VI (dibenzyl-14-crown-4), used in the CW-type ISE in this study, is not the highest-performing candidate in terms of the detection range when compared to other crown ether derivatives^26^ or other ionophores, such as lithium ionophore VIII.^27^ Thus, this observation does not imply that the detection range of the SB-ISE is inherently superior to that of the CW-ISE. The LOD of the SB-ISE is likely influenced by the trace dissolution of Li^+^ at the LATP surface.

At a lithium activity (*a*_Li_) of 0.1, the potential observed for the SB-type ISE was lower than that of the CW-type device. This discrepancy is attributed to the presence of lithium ions in the LiFePO_4_/FePO_4_ layer (from LiFePO_4_ and LiTFSA), which causes increased activity of Li ions in the composite layer (*a*_in_). Although *a*_in_ cannot be defined for CW-ISEs (*a*_in_ ≃ 0), an increase in *a*_in_ would lead to a lower membrane potential.^20,28^ The membrane potential (*E*_M_) is given by
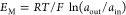
1where *R* is the gas constant, *T* is the absolute temperature, *F* is the Faraday constant, and *a*_out_ is the activity of the target ion outside the membrane.

The device-to-device potential deviation decreased markedly from approximately ±24 mV for the CW-type ISE to ±4 mV for the SB-type ISE. The substantial improvement in the reproducibility of the SB-type ISE is attributed to the use of LiFePO_4_/FePO_4_ as the solid contact layer; this material undergoes a two-phase reaction to provide a stable potential plateau, minimizing the potential drift.^20^ Employing the blend of LiFePO_4_ and FePO_4_ contributes to the high stability and reproducibility of the system as the internal reference electrode via the following two-phase reaction:^16^
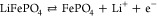
2

Furthermore, a consistent concentration of Li^+^ (from LiFePO_4_ and LiTFSA) within the solid-contact layer plays a crucial role in minimizing the variation in the activity inside the membrane (*a*_in_), thereby decreasing the potential deviation between different devices.^28^ This exceptionally low device-to-device variability is particularly critical for developing calibration-free ISEs, ensuring reliability and consistent ISE performance, without requiring individual calibration.^17^

Figure 2a illustrates the selectivity of the SB-ISEs evaluated using the separate solution method.^30^ Typically, a logarithmic selectivity coefficient (log*K*^pot^) of less than −2 is indicative of adequate selectivity. The SB-type ISE achieved a log*K*^pot^_Li_ of less than −2 for K^+^, Mg^2+^, and NH_4_^+^. However, only Na^+^ exhibited a log*K*^pot^_Li, Na_ greater than −1, suggesting insufficient selectivity. This can be attributed to the use of LATP with the Li^+^-substituted NASICON structure.^31^ Being inherently sodium-ion conductors, NASICON structures potentially allow some Na^+^ to permeate. Anticipated solutions to mitigate this issue include employing alternative Li^+^-conducting solid electrolytes, such as garnet or perovskite materials,^21^ although thin films of these materials are not yet widely commercialized. Figure 2b presents the long-term potential stability of the fabricated ISEs (three replicates each of CW- and SB-type ISEs) in a 0.01 mol dm^–3^ LiCl solution. For all three CW-type ISEs, a potential drift exceeding 100 mV was observed, particularly during the initial days of testing. This shift is likely due to the gradual permeation of the analyte solution into the organic ionophore-based ISM, resulting in increased *a*_in_, according to Equation 1, and the resultant decrease in the membrane potential. This trend aligns with the previously reported observation of a gradual potential decrease (>100 mV) for CW-type ISEs, with significant shifts occurring in the early stages of testing.^16^ In contrast, the SB-type ISEs exhibited minimal potential variations, ranging only between −3 and 6 mV over 17 d. This minimal deviation is notably within the expected range of potential variation of the Ag/AgCl reference electrode used in these measurements. Unlike the CW-type ISEs, which predominantly exhibited potential drifts in the negative direction, the varied direction of the potential shift in the SB-type ISEs underscores the absence of permeation of the analyte solution into the LATP plate. Water layer tests (Figure S1) further confirmed that the LATP plate prevents water penetration, precluding the formation of a water layer.

**Figure 2:**
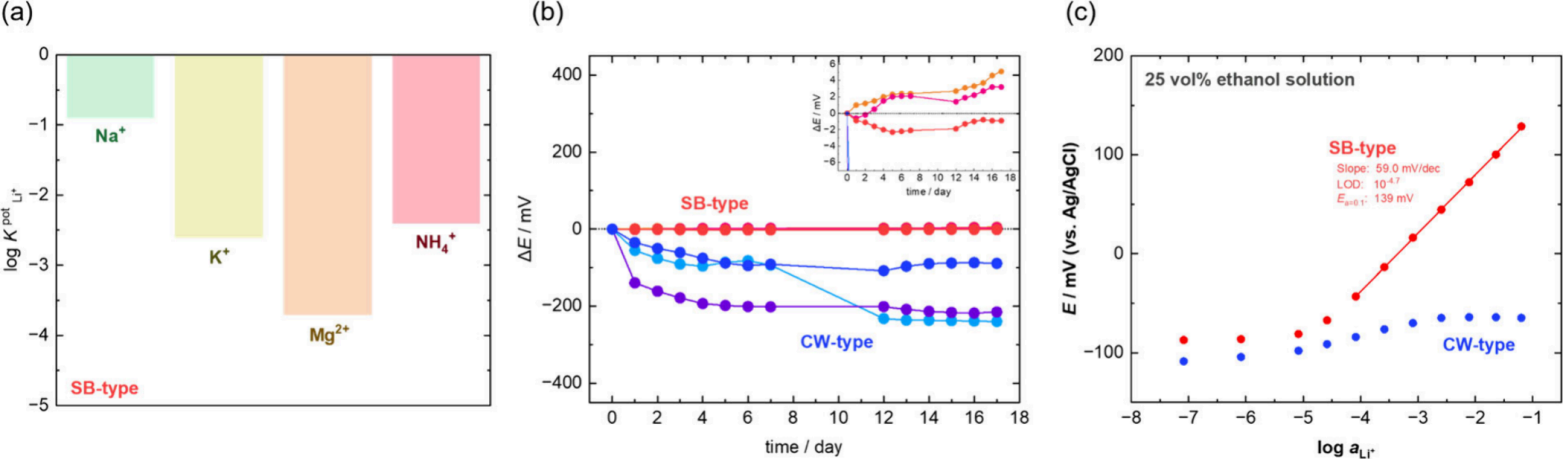
(a) Selectivity coefficient measurement for a solid-battery-type ion-selective electrode (SB-ISE) against Na^+^, K^+^ Mg^2+^, and NH_4_^+^ species measured by a separate solution method in aqueous LiCl (*a*_Li_ = 0.1) and NaCl, KCl, MgCl_2_, and NH_4_Cl solutions (*a*_*x*_ = 0.1).^30^ (b) Long-term potential stabilities of CW- and SB-ISEs stored in a buffered 0.01 mol dm^–3^ LiCl solution. ISEs were stored in the same solution during 8–11 d. (c) Calibration plots of the SB- and CW-ISEs examined in 25 vol % ethanol containing a buffered LiCl solution.

Considering that unlike plasticized poly(vinyl chloride) (PVC) membranes, LATP is not likely to deteriorate when exposed to organic solvents, we compared the potential responses of LATP and PVC membrane electrodes in mixed water–alcohol solutions containing Li^+^. Figure 2c shows the Nernst plots obtained in an aqueous solution containing 25 vol % of denatured ethanol. The CW-type ISE demonstrated a negligible potential response to varying Li^+^ concentrations, likely due to the dissolution of the organic ionophores in the ethanolic solution. In contrast, the SB-type ISE maintained a Nernst slope of 59.0 mV dec^–1^ because of the stability of LATP in organic solvents. This finding highlights the potential of the SB-type ISE in measuring ion concentrations in alcoholic beverages and foods containing alcohol, which is not feasible with conventional organic ionophores. Furthermore, the high stability of the inorganic LATP membrane may also enable it to effectively resist biofouling in real sample environments. Despite its promising performance in ethanol solutions, the SB-ISE exhibited a relatively high resistance of ∼10^4^ Ω cm^2^, as shown in Figure S2, whereas the LiFePO_4_/organic ionophore-based ISE showed a lower resistance of ∼10^3^ Ω cm^2^.^16^ The greater resistance of the SB-ISE may originate from the comparatively thick LATP plate (150 μm) used in this study and/or the interfacial resistance at the LATP/aqueous solution, the LATP/LiFePO_4_–FePO_4_–PEO-LiTFSA electrode, or others. However, utilizing a thinner solid electrolyte plate and/or adopting thin-film deposition techniques, such as physical vapor deposition, can effectively decrease this resistance, potentially enhancing the performance and applicability of the SB-ISE in various analytical scenarios. Although not identical, a similar concept can be observed in the relationship between fluoride shuttle batteries^32^ and fluoride ISEs,^33^ which have historically employed solid disks as ISMs.^34^ Notably, this study developed Li-ISEs inspired by Li-ASSBs, whereas the F-shuttle batteries were designed by drawing inspiration from F-ISEs, highlighting a historical reversal in these relationships.^35^

In conclusion, we developed an all-solid-state lithium ISE inspired by the ASSB design. The proof-of-concept of this design can be extended to other ISEs by employing solid-state ion conductors. The fabricated ISE utilizes a solid electrolyte, i.e., LATP, which has a Li^+^-transference number of 1 and thus exhibits membrane potential as the ISM. LATP was placed over LiFePO_4_/FePO_4_, a conventional positive electrode material that served as the internal reference electrode. The fabricated SB-type ISE demonstrated low potential variability across devices and exhibited long-term stability. Remarkably, the SB-type ISE performed reliably in alcohol-containing aqueous solutions, whereas the conventional ISE failed because of the elution of organophilic ionophores into the alcoholic solution. The findings of this study are crucial not only from the perspective of analyzing ion concentrations in alcoholic food products but also for gaining insights into the development of ISEs for a broad range of ions using similar configurations using other ion conductors (e.g., Na^+^ and K^+^ conductors). Such innovations highlight the potential of the cross-application of battery technology and analytical sensor development.

## Supporting Information Available

Experimental details, results of water layer tests on the solid battery-ISE system, and Nyquist plots (PDF)
