# Supplementary material for: All-Solid-State Ion-Selective Electrode Inspired from All-Solid-State Li-Ion Batteries
Source: Anal Chem. 2025 Feb 26;97(9):4819–23. doi: 10.1021/acs.analchem.4c06470 (PMC11912135; doi:10.1021/acs.analchem.4c06470)
Supplement: Supplementary file 1 — ac4c06470_si_001.pdf [file ac4c06470_si_001.pdf]

Supporting Information

All-Solid-State Ion-Selective Electrode Inspired from All-Solid-State Li-ion Batteries

Ryoichi Tatara,[†§] Yuki Shibasaki,[†] Daisuke Igarashi,[†] Hiroyuki Osada,[ζ] Kazuma Aoki,[ζ] Yusuke Miyamoto,[ζ] Toshiharu Takayama,[ζ] Takahiro Matsui,ζ Shinichi Komaba*[,†]

[†]Department of Applied Chemistry, Tokyo University of Science, 1-3 Kagurazaka, Shinjuku, Tokyo 162-8601, Japan; *komaba@rs.tus.ac.jp

[ζ]New Applications Research Center, KOA CORPORATION, 1633-28, Kitahara, Minami-minowa, Kamiina, Nagano, 399-4511, Japan

Present Address for [§]R.T.: Department of Chemistry and Life Science, Yokohama National University, Yokohama, Kanagawa, 240-8501, Japan

Table of Contents

-Experimental Methods

-Table S1. Extended Debye–Hückel constants for Li^{+}, Na^{+}, K^{+}, Mg^{2+}, and NH_4^{+}

-Figure S1. Results of water layer tests on the solid battery-ISE system in 0.01 mol dm^{-3} aqueous LiCl and KCl solutions.

-Figure S2. Nyquist plots of the solid battery-ISE in a 0.01 mol dm^{-3} LiCl buffer solution obtained under OCP conditions.

-References

EXPERIMENTAL METHODS

Materials

$LiFePO_4$ (Carbon coated, Hohsen Corp.), $Li_{1+x+y}Al_x(Ti, Ge)_{2-x}Si_yP_{3-y}O_{12}$ (LATP; LICGC™ AG-01, φ19 mm, 150 µm thick, OHARA INC.), lithium bis(trifluoromethanesulfonyl)amide (LiTFSA; >99.7%, Kanto Chemical Co., Inc.), LiCl (anhydrous, >99.0%, Nacalai), NaCl (>99.5%, Kanto Chemical Co., Inc.), KCl (>99.5%, Wako Chemicals), $MgCl_2 \cdot 6H_2O$ (>98.0%, Wako Chemicals), NH_4Cl (>99.5%, Wako Chemicals), tris(hydroxymethyl)aminomethane (>99.0%, TCI), acetylene black (AB, 50% compressed, Strem Chemicals), single-walled carbon nanotube dispersion with poly(vinylidene fluoride) (SWCNT/PVdF; TUBAL™ BATT), poly(vinylidene fluoride) (PVdF; #1100, Kureha Corporation), dibenzyl-14-crown-4 (lithium ionophore VI, >98.0%, Dojindo Laboratories, Inc.), poly(vinyl chloride) (PVC; Wako chemicals), 2-nitrophenyloctylether (NPOE; Wako chemicals), potassium tetrakis(4-chlorophenyl)borate (KTCPB; Aldrich), poly(ethylene oxide) (PEO; Mv ~600,000, Aldrich), *N*-methyl-2-pyrrolidinone (NMP; Kanto Chemical Co. Inc., anhydrous, >99.0%, $[H_2O]$: <50 ppm), tetrahydrofuran (THF; >99.7%, Kishida Chemicals), and ethanol (Wako Chemicals, >86%, denatured with 2-propanol) were used as received. Deionized water with a conductivity of <1.0 µS cm^{-1}, obtained using a purification system (Purelite, PRA-0015, Organo), was used in all experiments.

Electrode and membrane preparation

$LiFePO_4$ powder was dispersed in a 0.1 M aqueous $Na_2S_2O_8$ solution and stirred for 48 h. It was then collected via filtration and further washed with deionized water, followed by drying at 80 °C in ambient air. The composition of the resulting product was determined to be $Li_{0.011}FePO_4$ through inductively coupled plasma optical emission spectroscopy (ICP-OES). For simplicity, this sample is hereafter denoted as $FePO_4$. The carbon content of the carbon-coated $LiFePO_4$ was ignored in this study.

To prepare a composite electrode, $LiFePO_4$ and $FePO_4$ powders were mixed with AB, LiTFSA, PEO, and SWCNT/PVdF mixture dispersed in NMP using a planetary mixer (ARE-310, Thinky), and the obtained slurry was coated onto Al foil (20 µm thick, Hohsen). The mixing ratio of $LiFePO_4$:$FePO_4$:AB:LiTFSA:PEO:SWCNT:PVdF was 25.47:25.47:16.60:25.47:6.37:0.25:0.38 in weight percent, unless otherwise noted. The obtained

$LiFePO_4/FePO_4$ composite sheet was dried overnight at 60 °C. Then, the $LiFePO_4/FePO_4$ composite layer was covered with an LATP plate using a sample holder (AE9-4, EC Frontier). The so-fabricated ion-selective electrode (ISE) was maintained overnight at 80 °C to melt the PEO in it for improving the contact between the LATP and $LiFePO_4/FePO_4$ layers.

A coated wire (CW)-type ISE was prepared by directly drop-casting an organic ion-selective membrane (ISM) onto a Pt disk electrode (1 mm diameter embedded in epoxy resin). Dibenzyl-14-crown-4 (lithium ionophore VI), PVC, NPOE, KTCPB, and THF were used as the Li^+ ionophore, membrane matrix, plasticizer, lipophilic anion, and casting solvent, respectively, to prepare the Li^+-ISM.[1] The NPOE:ionophore:PVC:KTCPB mixture (65.5:0.9:33.3:0.3 (wt%)) was completely dissolved in THF (1.5 mL of NPOE and appropriate amounts of ionophore, PVC, and KTCPB were mixed using 7.5 mL of THF), and 10 μL of the above mixture was drop-casted for five times to cover the entire surface of the Pt electrode.[2] The as-obtained CW-ISE was dried at ambient temperature and pressure for 24 h and aged in a 0.01 mol dm^{-3} aqueous LiCl solution for another 24 h, before being rinsed with deionized water.[3] **Figure 1a-d** presents the schematic of the fabricated ISE.

Electrochemical measurements

Unless otherwise specified, all electrochemical measurements on the prepared electrodes were performed at room temperature using a potentiostat (HZ-5000, input impedance of $>10^{11}$ Ω, Meiden Hokuto Corp.). For electrochemical impedance spectroscopy (EIS), an HZ-5000 instrument was connected to a frequency response analyzer (5080, NF Electronic Instruments). Long-term potential stability tests were conducted using a voltage data-logger system (NR-500 with NR-TH-8, input impedance of $>10^7$ Ω, Keyence). The open-circuit potential (OCP) was measured in 0.01 mol dm^{-3} aqueous tris(hydroxymethyl)aminomethane solutions with different LiCl concentrations using a two-electrode configuration against double junction Ag/AgCl electrodes; a saturated KCl aqueous solution was used as both inner and outer filling solutions.[2, 3] The concentration of the test solution was increased incrementally by adding appropriate amounts of the aqueous LiCl solution during OCP measurements. A 0.01 mol dm^{-3} tris(hydroxymethyl)aminomethane solution was used as a buffer, except in selectivity and water layer tests. The buffer solution was employed to enhance the ionic conductivity of the sample solution, particularly

at a very low Li^+ concentration. The extended Debye–Hückel equation (Equation S1) was used to convert the LiCl concentration (c_{LiCl}) to Li^+ activity (a_{Li+}):

$a_{Li+} = \gamma_{\pm} c_{LiCl}$ and $\log \gamma_{\pm} = [-AI^{1/2}/(1+BI^{1/2})] + CI$ (Equation S1)

where A, B, and C are constants, as listed in **Table S1**, and I is ionic strength.[4] For ethanol-containing solutions, Debye–Hückel constants A, B, and C were assumed to remain unchanged. Other electrochemical tests were conducted using a three-electrode configuration with a double-junction Ag/AgCl reference electrode and Pt wire counter electrode. EIS was performed at the OCP with a sinusoidal amplitude of 100 mV in the frequency range of 100 kHz to 10 mHz.

Table S1. Extended Debye–Hückel constants for Li^+, Na^+, K^+, Mg^{2+}, and NH_4^+

	A	B	C
Li^+	0.5108	1.3354	0.1079
Na^+	0.5108	1.4255	0.02626
K^+	0.5108	1.2796	0.00393
Mg^{2+}	0.5108	1.7309	0.05195
NH_4^+	0.5108	1.3061	0.00051

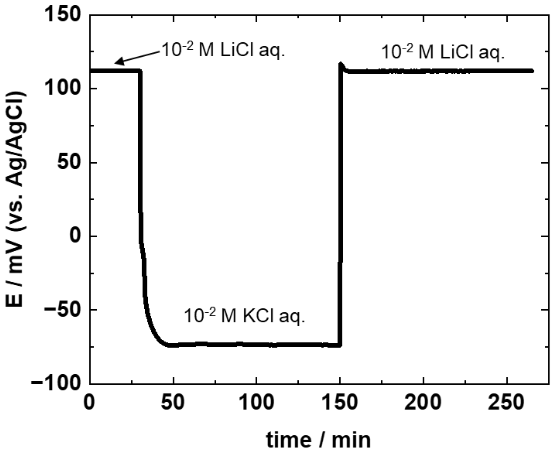

Figure S1. Results of water layer tests on the solid battery-ISE system in 0.01 mol dm^{-3} aqueous LiCl and KCl solutions.

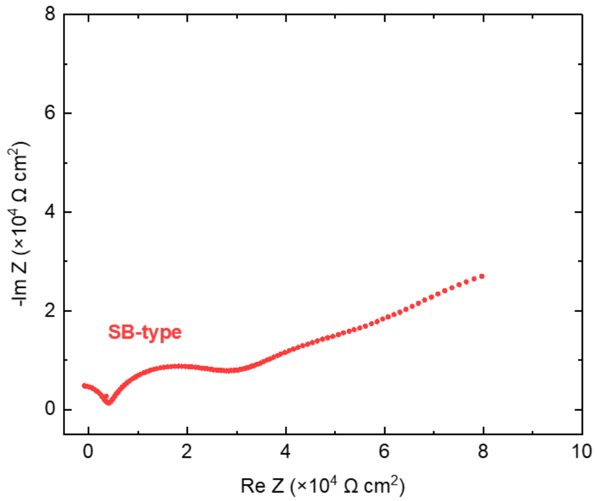

Figure S2. Nyquist plots of the solid battery-ISE in a 0.01 mol dm^{-3} LiCl buffer solution obtained under OCP conditions.

References

(1) Bakker, E.; Bühlmann, P.; Pretsch, E. Polymer Membrane Ion-Selective Electrodes -What are the Limits? *Electroanalysis* **1999**, *11* (13), 915-933.

(2) Komaba, S.; Akatsuka, T.; Ohura, K.; Suzuki, C.; Yabuuchi, N.; Kanazawa, S.; Tsuchiya, K.; Hasegawa, T. All-solid-state ion-selective electrodes with redox-active lithium, sodium, and potassium insertion materials as the inner solid-contact layer. *Analyst* **2017**, *142* (20), 3857-3866.

(3) Tsuchiya, K.; Akatsuka, T.; Abe, Y.; Komaba, S. Design of all-solid-state chloride and nitrate ion-selective electrodes using anion insertion materials of electrodeposited poly (allylamine)-MnO_2 composite. *Electrochim. Acta* **2021**, *389*, 138749.

(4) Meier, P. C. Two-parameter Debye-Hückel approximation for the evaluation of mean activity coefficients of 109 electrolytes. *Anal. Chim. Acta* **1982**, *136*, 363-368.
